# Genetics and educational attainment

**DOI:** 10.1038/s41539-017-0005-6

**Published:** 2017-02-01

**Authors:** David Cesarini, Peter M. Visscher

**Affiliations:** 1 Department of Economics, New York University, New York, NY 10012 United States; 2 Institute for Molecular Bioscience, University of Queensland, Brisbane, QLD 4072 Australia; 3 Queensland Brain Institute, The University of Queensland, Brisbane, QLD 4072 Australia

## Abstract

We explore how advances in our understanding of the genetics of complex traits such as educational attainment could constructively be leveraged to advance research on education and learning. We discuss concepts and misconceptions about genetic findings with regard to causes, consequences, and policy. Our main thesis is that educational attainment as a measure that varies between individuals in a population can be subject to exactly the same experimental biological designs as other outcomes, for example, those studied in epidemiology and medical sciences, and the same caveats about interpretation and implication apply.

## Introduction

Human geneticists interested in traits that vary between people in a population employ a number of experimental designs to ask specific questions. Broadly, the questions that are addressed are as follows:

i. Does empirical evidence exists for genetic factors that underlie variation in the population?
ii. What are the relative contributions of genetic and environmental factors to the observed variability?
iii. How many genes in the genome are involved in accounting for a variation attributed to a genetic variation, and what are the effect sizes at those genes?
iv. Are the genetic effects also associated with other traits?
v. Why is a genetic variation present in the current population (which is the same as asking how the variation in maintained by evolution)?

Many other disciplines wish to address questions that stem from genetic findings. Those questions can be summarized as “What can we do with this information?” A biologist may wish to understand the function of a particular genetic mutation, a medical researcher may wish to create therapeutics to cure a disease, and an epidemiologist may wish to identify a modifiable risk factor and influence health policy. In this review/perspective, we address recent findings about biology and education, and how these findings could be useful for researchers and practitioners working in education, science, and learning.

Behavior–genetic studies of twins and adoptees consistently find genetic factors accounting for at least a modest share (up to 40%) of differences in educational outcomes across individuals in developed, Western countries,^1,2^ but it only recently became feasible to start identifying some of the DNA variants responsible for this genetic variation.^3^ In this review article, we discuss how, if at all, such advances could constructively be leveraged to advance research on education and learning.

To set the stage, we begin by reviewing evidence from behavior–genetic studies on the heritabilities of various traits related to learning, memory, and education. A heritability is a statistical parameter describing the proportion of variation in a population that is accounted for by genetic factors.^4^ We review the methodology of these studies and their findings, and we discuss what can and cannot be concluded from heritability estimates of educational phenotypes, such as years of schooling, measures of scholastic achievement, psychological characteristics, learning disabilities, or neurodevelopmental disorders.

Our ability to reliably detect specific genetic polymorphisms associated with various behavioral traits has increased dramatically in recent years.^3^ An important lesson emerging from studies to date is that single polymorphisms almost always have small effects—and for this reason, their detection requires very large samples.^5^ As larger and larger discovery samples become available, the number of credibly established associations with a wide range of outcomes will continue to grow. The larger samples will also allow researchers to construct increasingly powerful predictors, called polygenic scores (PGSs), from genetic information.

Finally, we also discuss how these and future findings from this literature may be put to productive use in research on education and learning. We organize our discussion around three broad classes of potential contributions.

First, controlling of genetic factors in empirical research will become increasingly possible, thus strengthening the credibility of many research designs (for example, by improving the statistical power a randomized controlled trial to estimate a treatment effect). Currently, a PGS for educational outcomes has a modest predictive power (*R* ^2^ ≈ 7%, implying a correlation between predictor and outcome of ~0.26), but this number is likely to increase substantially in the years ahead.

Second, and more speculatively, insights into the genetics of educational outcomes—and their many precursors in the form of intelligence, personality dimension, dyslexia or attention problems—may yield biological insights that provide guidance for drug-discovery efforts or new directions for our theoretical understanding of genetic mechanisms of learning and memory. The discovery of genes associated with educational outcomes may also provide insights into genetically correlated outcomes, such as the risk of dementia.

Third, and most speculatively, the identification of genetic factors that influence educational outcomes may point to modifiable channels through which genes influence scholastic outcomes. In some cases, such information may allow parents to take preemptive actions tailored to the child’s specific needs that would not have been possible absent the genetic information.

## Heritability

Heritability is a population parameter that can be estimated using different experimental designs. Until technological advances facilitated the direct measurement of DNA variants, the heritabilities of various humans were mostly estimated by comparing the resemblance of twins, adoptees, and other pairs of relatives.

To explain heritability, a simple and highly stylized causal model is useful; our treatment follows Benjamin *et al.* ^6^ We assume the genetic variants influencing the outcome of questions are located at *J* separate locations (“loci”) in the genome. At each locus, individuals are endowed with two alleles, one inherited from the mother and one from the father. We can arbitrarily designate one of these to be the reference allele, and define person *i*’s genotype at locus *j*, *x*_*ij*_,  by the number of reference alleles person *i* is endowed with (because we make the simplifying assumption of only two alleles, this number is always equal to 0,1 or 2).

We assume the following causal model for person *i*’s outcome:

1 $${Y_i}={\underbrace{\sum \nolimits_{j=1}^{J}{x_{ij}}{\beta_j}}\limits_{\equiv G_i}+{U_i},}$$

where *j* indexes the loci, *β*_*j*_ is the causal impact of an additional copy of the reference allele at locus *j* (this impact need not be the same across time and space), and *U*_*i*_ is an environmental variable. If *Y*_*i*_ is normalized so its standard deviation is 1, and *G*_*i*_ and *U*_*i*_ are uncorrelated, the total variance in *Y*_*i*_ is the sum of two components: a genetic factor (*h* ^2^≡*var*(*G*_*i*_)) and a non-genetic factor (*u* ^2^≡*var*(*U*_*i*_)). The parameter *h* ^2^—known as (narrow sense) heritability—is simply the *R* ^2^ from the population regression of the outcome on the *J* genotypes.

One can make inferences about the *h* ^2^ of a trait without knowing the specific genes responsible for the heritable variation. Prior to the widespread availability of molecular data, most such efforts have relied on comparisons of the resemblance in the observable characteristics (“phenotypes”) of various pairings of relatives who vary in their degree of environmental and genetic resemblance. In these studies, a commonly made assumption is that the distributions of *G*_*i*_ and *U*_*i*_ are the same across all types of siblings. Randomly ordering the two members of a sibling pair and denoting the second member of a pair by a prime, most heritability estimates are based on identifying conditions that have the general form:

2 $${\rho _{\rm YY'}^{\rm k}}=E({Y_i}{Y_i'})={\rho _{\rm GG'}^{\rm k}}{h^2}+{\rho _{\rm UU'}^{\rm k}}{u^2}.$$

On the left-hand side of Eq. 2 is the phenotypic correlation, whose sample analog is easily estimated by drawing a random sample of sibling pairs of type k and calculating the pairwise correlation between their outcomes. The methodology of studies in “behavior genetics” is fundamentally about using estimated phenotypic correlations ${\hat{\rho} _{\rm YY'}}$ for various types of siblings to infer population parameters such as *h* ^2^ and *u* ^2^. In general, assumptions about ${\rho _{\rm GG'}}$ are often based on population genetic theory,^7^ whereas inferences about ${\rho _{\rm UU'}}$ are based on whether the siblings of a given type were raised in the same household.

For example, most twin studies compare the resemblance of monozygotic twins reared apart (*k* = MZT) with the resemblance of dizygotic twins reared together (*k* = DZT). In studies attempting to estimate heritability from twin data, a commonly made assumption is that Eq. 2 holds for both types of twins, with ${\rho _{\rm GG'}^{\rm MZT}}=1$, ${\rho _{\rm GG'}^{\rm DZT}}=0.5$ and ${\rho _{\rm UU'}^{\rm DZT}}={\rho _{\rm UU'}^{\rm MZT}}\equiv {\rho _{\rm UU'}^{\rm T}}$. If these conditions hold, it is straightforward to verify that $2({\rho _{\rm YY'}^{\rm MZT}}-{\rho _{\rm YY'}^{\rm DZT}})={h^2}$. An estimator of heritability is the sample analog of this moment condition, also known as Falconer’s estimator: ${\hat{h}^2}=2({\hat{\rho}_{\rm YY'}^{\rm MZT}}-{\hat{\rho}_{\rm YY'}^{\rm DZT}}).$

**Fig. 1:**
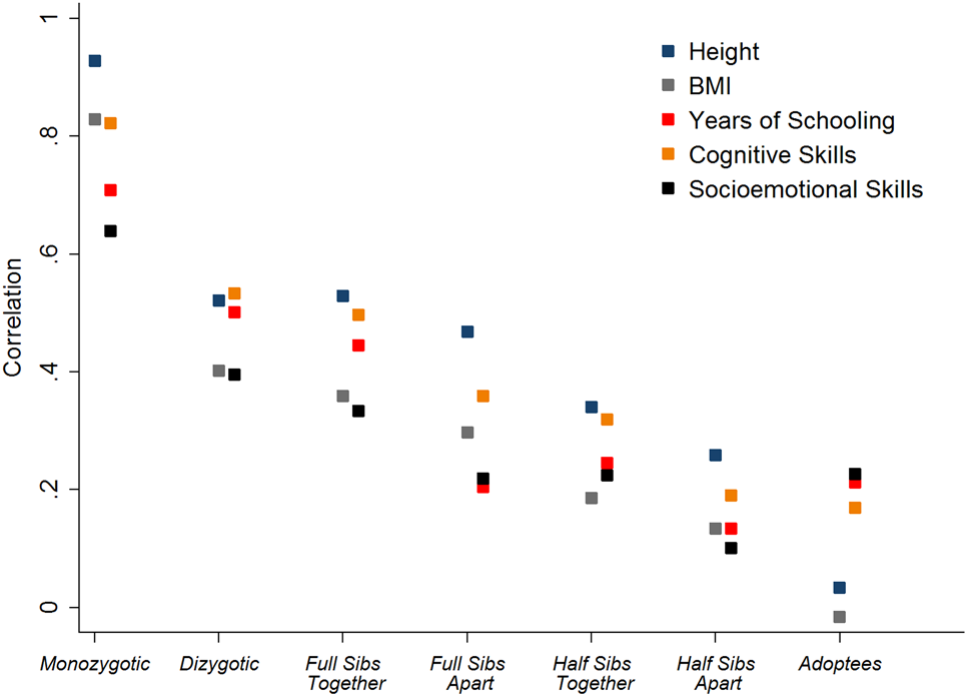
Sibling Correlations for Behavioral Traits. This figure displays sibling correlations for five traits measured in a large sample of Swedish brother pairs born 1951–1970. All outcomes except years of schooling are measured at conscription, around the age of 18. For details on sample construction and variable definitions, see chapter 3 in Cesarini.^10^ The sample sizes vary by outcome, but the minimum number of pairs per sibling type is MZT = 1,154; DZT = 1,601; FST = 151,789; FSA = 1,033; HST = 4,880; HSA = 11,566; ADO = 643. Because the sample sizes are large, all correlation coefficients are precisely estimated. The standard error of a correlation coefficient estimated with *N* pairs of siblings is approximately $(1-{\hat{\rho }}^{2})/\sqrt{N}$. For example, the approximate standard error of the 72% estimate reported in the full text (full siblings reared apart) is $2\times (1-{\hat{\rho }}_{{\rm{FSA}}}^{2})/\sqrt{N}=2\times (1-{0.359}^{2})/\sqrt{1033}\approx 0.054$.

To illustrate some canonical findings from the literature, Fig. 1 displays phenotypic correlations for three education phenotypes—years of schooling, cognitive skills, and socioemotional skills for—and two anthropometric phenotypes—height and body mass index – in seven types of Swedish sibling pairs who differ in their genetic and environmental relatedness. The correlations are computed using administrative data covering all Swedish brother pairs born between 1951 and 1970, and have been previously reported.^8–10^ With the exception of years of schooling, all data are from Swedish conscription data, and measured around age 18 (the year of enlistment).

Our measure of cognitive skills is derived from the conscript’s score on four cognitive tests (synonyms, spatial skills, inductions and technical comprehension) and is highly correlated with what is sometimes referred to as general intelligence.^11^ Our measure of socioemotional skills is based on a professional military psychologist’s assessments of the conscript’s ability to function in the military, with higher scores assigned to recruits, which the psychologist perceives as independent, emotionally stable, able to function in a group and willing to take on responsibility.^12^ Lindqvist and Vestman^12^ document that the variable (which they call noncognitive ability) is a much stronger predictor of labor market outcomes than the personality dimensions measured by standard personality scales.

In these analyses, two brothers are classified as “reared apart” if they lived in separate households during every census undertaken before the age of 18, and “reared together” otherwise. We use information about biological parents to classify siblings reared in the same household as full brothers (same biological parents), half-brothers (share one biological parent), and adoptees (share no biological parents but reared in the same household). Two broad patterns are evident from Fig. 1 First, for all five traits, the phenotypic resemblance of pairs of siblings reared together increases with genetic relatedness. Second, holding constant genetic relatedness, siblings reared in the same household are usually more similar than siblings reared in separate households.

Until recently, data such as those in Fig. 1 were the primary source of information about the heritability of various traits. To illustrate how sibling correlations can be used to decompose phenotypic variation, applying Falconer’s formula to the correlations gives ${\hat{h}^2}=2({\hat{\rho_{\rm YY'}^{\rm MZT}}}-{\hat{\rho}_{\rm YY'}^{\rm DZT}})=2(0.822-0.534)= 58 \%$ for cognitive skills and ${\hat{h}^2}=2({{\hat\rho _{\rm YY'}^{\rm MZT}}}-{\hat{\rho} _{\rm YY'}^{\rm DZT}})=2(0.928-0.521)=81 \%$ for height. With seven sibling types, many other estimators are available. For example, if we assume that in full siblings reared apart, ${\rho _{\rm GG'}^{\rm FSA}}=0.5$ and ${\rho _{\rm uu'}^{\rm FSA}}=0,$ then $2{\rho_{\rm YY'}^{\rm FSA}}={h^2}$. The analogy principle then suggests the estimator ${\hat{h}^2}=2{\hat{\rho }_{\rm GG'}^{\rm FSA}}$ , giving us ${\hat{h}}^{2}=2\times 0.359=72 \%$ for cognitive skills and ${\hat{h}}^{2}=2\times 0.468=94 \%$ for height. In practice, if feasible, it is almost always advisable to use an estimate that incorporates information from as many different sibling types as possible (not just twins). Most importantly, information about additional sibling types provides identifying variation that can be used to estimate richer models that relax (or test) some of the potentially problematic assumptions underlying Falconer’s formula.

Though the data in Fig. 1 are quite representative of findings in the behavior–genetic literature, it bears emphasizing that heritabilities are population-specific parameters (not universal constants). Heritabilities can (and do) vary across time and space and for some traits, they can also vary in interesting ways over the lifecycle. For example, one of the most robustly replicated findings from the behavior–genetic literature is that the heritability of cognitive skills rises gradually through childhood and adolescence.^13^

## (Mis)interpreting heritability estimates

What should we infer from data such as those plotted in Fig. 1? One common albeit mistaken inference is that since all the four traits studied appear to have substantial heritabilities, efforts to modify them through environmental interventions are doomed to failure.

It does not follow logically from the observation that when *u* ^2^ is low, the environmental interventions can be effective (nor does it follow from an observation that *u* ^2^ is large for some trait that it is straightforward to modify the trait through environmental channels; see Sacerdote^14^). For example, the provision of eyeglasses could yield large returns in a population where very few children have eyeglasses and the heritability of eye sight is near 100%.^15^

Jumping to policy conclusions from high heritabilities is problematic for a second, distinct reason. Genes may sometimes—perhaps often—influence complex outcomes through channels that are modifiable (channels that would be labeled “environmental” in common parlance but are part of the heritable variation in the standard decomposition). The fact that genes influence an outcome does not imply they must do so through some narrowly physiological process.^16^ Factors that may be important inputs to a person’s learning process—such as motivation, ability to concentrate, or time spent reading books—are all under some genetic influence. But the extent to which genetic risk factors for, say, concentration difficulties actually translate into worse scholastic performance is likely to depend in subtle ways on the design of the education system. To take an even starker example, the education system’s treatment of boys and girls has changed dramatically over the course of the 20th century, along with changes in norms and attitudes.

What then, should one conclude from data such as those in Fig. 1? A first conclusion is that most of the observed resemblance between genetically related siblings can ultimately be traced to their genes. This observation suggests to us that trying to understand the mechanisms through which genes influences complex outcome such as BMI or performance on a cognitive test may be a worthwhile enterprise. A second conclusion is that it should be possible, at least in principle, to predict a range of scholastic outcomes from genetic data, with sufficient information. As we argue below, such a possibility may prove valuable to many researchers in the coming years.

Data such as those in Fig. 1 are sometimes used to justify much stronger conclusions than those we consider appropriate. Canonical findings from the literature on the heritability of cognitive ability have historically been used to argue for earlier ability tracking and, more recently, calls for “genetically sensitive” schools.^17^ Most schooling systems eventually separate children into different tracks (e.g., vocational or academic), often using past scholastic performance as a major screening device, and we suspect the proposition that some sort of screening at some point is desirable, which has broad agreement. But for the reasons articulated by Goldberger and Jencks, we do not believe any obvious relationship exists between the data pictured in Fig. 1 and issues such as the optimal timing of ability tracking.

Calls for “genetically sensitive” school are sometimes met by concerns that advances in genomics will be used to justify denying children educational opportunities as opposed to help children better realize their potential. All schooling systems already have at least some features that can be characterized as “genetically sensitive” in the sense that heritable child characteristics (such as school grades) are used to assign children to the environments. Children with poor eyesight are (hopefully!) supplied with glasses; children with low grades are more likely to be required to retake a grade level; children with learning disabilities are sometimes educated by specially trained teachers who rely on different pedagogies and teaching materials; eyesight, test scores, and virtually all learning disabilities have some degree of heritability.

Ultimately, what matters is whether these interventions generate benefits that can justify their costs, not whether the bad eyesight or learning disabilities are ultimately caused by genes. In our opinion, it is only to the extent that genetic information makes it possible to tailor more effective interventions that genetic data may be a useful supplement to systems already in place.

## The modern GWAS era

In the last decade, data on molecular-level genetic markers that differ across individuals, called single-nucleotide polymorphisms (SNPs), have become much more widely available as costs of measuring SNPs have plummeted. As a result, research has begun to identify specific SNPs that account for some of the heritable variation in anthropometric traits, common diseases, and, in a handful of cases, behavioral outcomes such as educational attainment or smoking.^3,9^ Today, most of these studies are hypothesis-free scans for associations between some outcome and millions of genetic variants (each of which is separately tested for association with the outcome). The large number of hypotheses tested means a variant is considered associated if the *p*-value for association is below the genome-wide significance threshold, 5 × 10^−8^.

In the early years of genome-wide association studies (GWASs), the fact that most of the studies published at that time had identified only a small number of SNPs was commonly (mis)interpreted as evidence that GWAS is a flawed approach. Within medical genetics, today it is increasingly understood that an ever-increasing number of SNPs associated with complex outcomes are being identified as the sample sizes have grown. This empirical regularity is illustrated in Table 1, which shows how the number of identified associations with education, height, and BMI has increased with large discovery samples.

**Table 1:** Sample size and number of genome-wide significant associations

Years of education			Height			Body-mass index		
Ref.	*N*	#Hits	Ref.	*N*	#Hits	Ref.	*N*	#Hits
^1^	8352	0	^4^	15,821	12	^9^	11,536	0
^2^	101,069	1	^5^	16,482	20	^10^	123,865	19
^2^	126,559	4	^6^	30,968	27	^11^	339,224	97
^3^	293,723	74	^7^	183,727	180			
^3^	405,072	162	^8^	253,288	697			

*Note*. Relationship between the size of the discovery sample and the number of approximately independent loci identified at the genome-wide significance level (“hits”) for three outcomes. 1. Benjamin *et alet al*.^22^ 2. Rietveld *et alet al*.^9^ 3. Okbay *et alet al*.^23^ 4. Lettre *et alet al*.^24^ 5. Weedon *et alet al*.^19^ 6. Gubjartsson *et alet al*.^18^ 7. Lango Allen *et alet al*.^20^ 8. Wood *et alet al*.^21^ 9. Liu *et al*.^25^ 10. Speliotes *et al*.^26^ 11. Locke *et al*.^27^

Consider the example of height. Early studies of height identified 10–20 SNPs at genome-wide significance,^18–20^ whereas more recent research,^21^ based on a sample of 253,000 individuals, identifies 697. The first genome-wide association study of educational attainment was conducted in a discovery sample of 101,069 individuals, and identified one significant association with years of schooling.^9^ The authors conducted the follow-up study in a discovery sample size of 293,723 individuals, and identified 74 loci associated with years of schooling completed. In a combined analysis of the discovery and replication samples (*N* = 405,072), the number of independent loci further increases to 162. Thus, educational attainment appears to follow a pattern that is qualitatively similar to medical and anthropometric traits.

Medical geneticists have developed methods to construct PGSs that can be used to exploit the joint effects of many genetic variants. Most commonly, such PGSs are constructed using some version of Eq. 1, albeit replacing the individual *β*_*j*_s (which are unobserved) with estimates obtained from an independent sample.^28^ For most complex traits, the combined explanatory power of the genome-wide significant associations uncovered so far is very modest, even if combined into a polygenic score. For example, the 162 loci, found to be associated with years of schooling jointly, explains less than 1% of variance across individuals; the analogous figures for BMI and height are 12.5 and 3%, respectively.

Following the publication of some of the earliest GWASs, the gap between the explanatory power of the variants identified and estimates from twin studies prompted a spirited debate on the causes of the “missing heritability”.^29–32^

Though the missing heritability seems unlikely to have a single explanation, researchers now broadly agree a substantial fraction of the heritability was not “missing,” but was rather hiding in the form of SNPs whose effects were so small they evaded detection even in discovery samples of hundreds of thousands of individuals. This consensus is based on several convergent lines of evidence, of which the qualitative patterns described in Table 1 is only one.

Most studies to date have found the predictive power of PGSs is maximized if the markers included to generate the scores are selected using a more liberal *p*-value threshold than genome-wide significance. The fact that prediction accuracy improves when variants that failed to reach genome-wide significance are added suggests many of these marginal associations represent true associations of genetic variants that will reach genome-wide significance in sufficiently large samples.

**Fig. 2:**
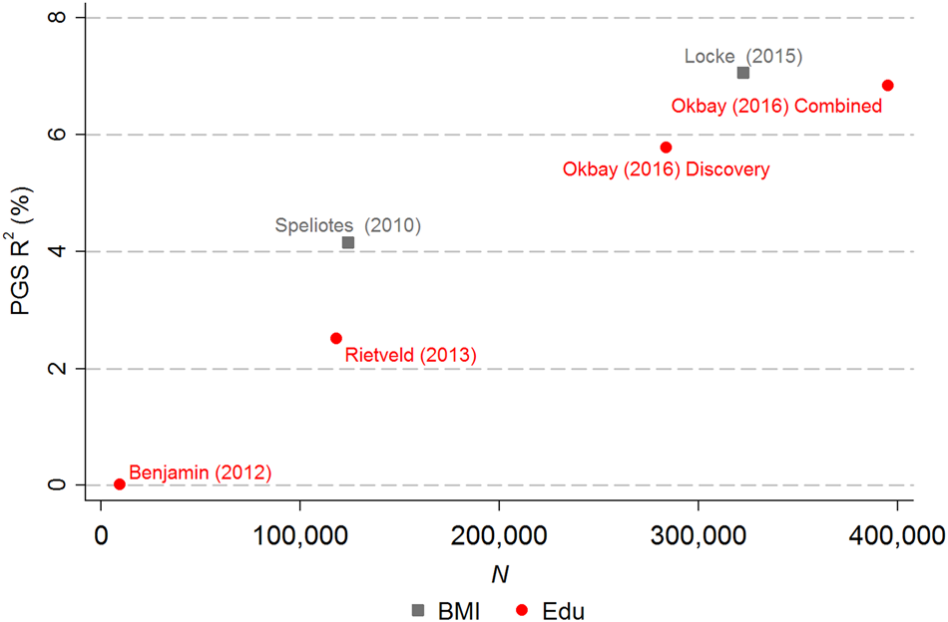
Predictive power of PGSs derived from weights estimated in discovery samples of various sizes. All dependent variables have been residualized on sex, age, and 10 principal components of the variance–covariance matrix of the genotype data. To avoid overfitting, all scores are based on meta-analyses that omit the HRS from the discovery sample.

Also consistent with the hypothesis of “hidden” heritability, the predictive power of PGSs has increased as larger and larger discovery samples have become available. Intuitively, larger samples enable constructing PGSs with greater predictive power, because the expected deviation between ${\hat{\beta }}_{j}$ and *β*_*j*_ falls as larger and larger samples reduce estimation error. Figure 2 illustrates this point for three phenotypes using data on genotyped respondents of European ancestry in the Health and Retirement Study (HRS). The *left panel* shows the results for body mass index BMI and years of schooling. An important interpretational caveat is that our analyses and projections are for samples of European ancestry. Because the original GWASs were conducted in samples of European ancestry, a PGS derived from the GWAS results would have substantially lower predictive power in non-European populations.^33^

For BMI and EduYears, PGSs with weights derived from the first large-scale GWASs (*N *≈ 100,000) explain around 3% of variation in independent samples. As sample sizes have increased to *N* ≈ 300,000 (BMI) and *N *≈ 400,000 (EduYears), the predictive power of PGSs has increased to about *R* ^*2*^ ≈ 7%. For height, the qualitative patterns are similar, but the level of predictive power is higher at all sample sizes (unsurprisingly, given that height is a more heritable trait).

**Fig. 3:**
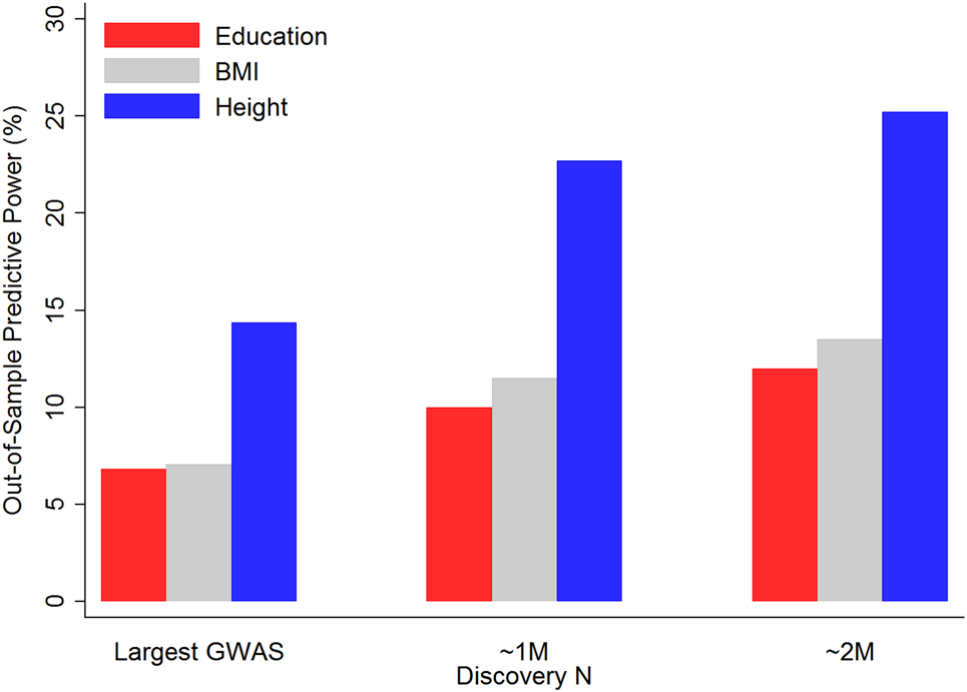
Predictive power of future PGSs. Currently attainable predictive power of PGSs (“Largest GWAS”) and projected explanatory power of future PGSs derived from discovery samples of 1M and 2M individuals (“~1M” and “~2M”). For each trait, we calculate ${h}_{{\rm{SNP}}}^{2}$ directly from Eq. 3. We assume M = 70,000, and using values of *R* ^2^ and *N* from the largest published GWAS of the trait (e.g., 6.9% and 405,000 for education). Implied SNP-based heritabilities are 15, 16 and 30%, respectively. Parameterizations based on estimates of SNP-based heritabilities (25, 25 and 50%) yield higher projections.

To be clear, for all three traits, a substantial gulf remains between the predictive power of the PGSs and the estimates of twin and family studies. The currently attainable degree of predictive power is roughly 15–20% of the behavior–genetic estimates implied by the correlations in Fig. 3. One important source of the gap is that the estimand in behavior–genetic studies is the proportion of variance explained by all genetic factors, including those not captured (“tagged”) by standard genotyping arrays currently used in GWASs. PGSs constructed from common variants therefore have a lower theoretical upper bound than the twin and family estimates. This bound, known as the SNP heritability, can be estimated given suitable SNP data.^29,34,35^ Published estimates suggest common variants account for about 50% of variation in human height,^21^ and around 25% of variation in traits such as BMI and educational attainment.^9,27^

## Taking stock

Our discussion so far has sought to describe and interpret the results from studies of educational attainment, BMI, and height. We focused on these phenotypes because very large GWASs have been conducted for all three, and because we believe many of the lessons from these studies are likely to apply generally to many other complex traits. As genotyping costs continue to plummet, researchers will likely be able to conduct GWASs of a wide range of behavioral and scholastic outcomes in discovery samples larger than a million individuals. For example, a dataset with detailed health information on around 500,000 genotyped British individuals is scheduled to be available to researchers in 2017; similar initiatives are underway in many other countries.

How might such data sets contribute to research on science on learning? We organize our discussion below around three broad classes of potential contributions.

## Controlling for confounding factors

### Predicting the predictive power of future PGSs

Figure 2 shows the prediction accuracy of PGSs has increased steadily as larger samples have become available. But how much predictive power can we realistically expect from future PGSs generated from discovery samples comprising millions of individuals? The expected *R* ^2^ of a polygenic score and the sample size used to generate the ${\hat{\beta }}_{j}$’s used as weights can be characterized analytically^36^ as

3 $${R}^{2}\approx {h}_{{\rm{SNP}}}^{2}{[1+\frac{M}{N{h}_{{\rm{SNP}}}^{2}}]}^{-1},$$

where ${h}_{{\rm{SNP}}}^{2}$ is the total proportion of variation explained by the measured genetic variants, *N* is the sample size used to estimate the weights, and *M* is a population-genetic parameter roughly equal to 70,000 in modern European populations. Because ${h}_{{\rm{SNP}}}^{2}$ can be estimated using the method of Yang *et al*. (2010), this formula can be calculated quantitatively. Figure 3 shows the predictive power currently attainable from the largest published GWAS for each of our three traits: education, BMI, and height, followed by conservative (for reasons described below) projections of the predictive power of future PGSs generated from discovery samples of 1M and 2M individuals. The projection suggests the predictive power of a polygenic score constructed using weights from a discovery sample of 2M individuals comes very close to the theoretical upper bound given by the SNP heritability.

Much of the uncertainty about the ultimately attainable predictive power stems from uncertainty about the SNP heritabilities. The projections in Fig. 3 are based on values of ${h}_{{\rm{SNP}}}^{2}$ calculated from Eq. 3 using actual values of *R* ^*2*^ and *N* from the largest meta-analysis of the trait available to date (e.g., *R* ^2^ = 6.9% and *N* = 405,000 in the case of education). These meta-analyses were performed by combining summary statistics from dozens of cohorts that vary across important dimensions, including the birth cohort of the genotyped respondents, the phenotype measures used, country, and exact genetic ancestry.

The resulting heritabilities (15/16/30% for education, BMI and height, respectively) are approximately one-third lower than the estimates based on the method of Yang *et al*.^29^ This discrepancy is likely at least in part due to: (i) phenotypic heterogeneity across cohorts included in the meta-analyses, (ii) genetic heterogeneity across cohorts, (iii) gene-by-environment interactions across cohorts, and (iv) undetected errors in the meta-analysis. Most of these sources of downward bias can be mitigated (and in some cases, eliminated altogether) if the discovery sample is restricted to a single, genetically and environmentally homogenous cohort. Therefore, with the advent of resources such as the UK Biobank, the numbers in Fig. 3 likely paint a picture of future prediction accuracy that errs on the side of pessimism.

### PGSs as control variables

PGSs for educational attainment might first prove valuable as control variables in randomized-control trials on the impact of an intervention on some scholastic outcome. Controlling for a score could improve the statistical precision of the study by reducing the amount of residual variance.

Education researchers frequently use randomized controlled trials to study the effectiveness of various interventions.^37^ Substantial uncertainty remains about the causal effects of many of these interventions, partly because many of the studies were conducted in small samples. As a result, credibly ruling out that any observed difference between the outcomes of treated and untreated subjects is due to chance fluctuations (as opposed to an effect of the intervention) is often difficult. The concern about small sample size applies especially to some of the most influential studies on the effectiveness of early childhood interventions, such as the HighScope Perry Preschool Study and the Abecedarian Project.

Rietveld *et al*.^9^ consider the decision problem of a hypothetical researcher whose objective is to maximize the statistical power to detect a treatment effect and who must choose between the following two options:

1. Conduct a randomized controlled study in a sample of *N*_X_ experimental participants, a proportion *p* of whom are randomly assigned the treatment. For all study participants, a set of baseline characteristics explaining 10% of outcome variance is available.
2. Conduct a randomized controlled study in a sample of *qN*_X_ experimental participants, a proportion *p* of whom are randomly assigned the treatment. For all study participants, a set of baseline characteristics explaining 10% of outcome variance is available. Additionally, a PGS whose incremental predictive power is X% is available.

For a range of values of *X*, the authors ask for what value of *q*, the investigator is indifferent between the two options. Clearly, the answer to this question is most interesting for a realistic choice of *X*. According to Fig. 3, a PGS constructed using summary statistics from a GWAS of 2,000,000 individuals has a prediction accuracy of around 12%. Rietveld *et al*.^9^ show (SOM Table S27) that under the assumption that the PGS and baseline characteristics are uncorrelated, the investigator should be indifferent between the two options at *q* = 0.87. Thus, the PGS in this example allows a cost-saving equivalent to reducing the sample by 13%. The cost savings are smaller if the baseline characteristics are correlated with the PGS, because in that case, the incremental explanatory power of the PGS will be smaller than 12%.

Yet, as Rietveld *et al*.^9^ note, for very expensive interventions, even a modest reduction in sample size may yield substantial cost savings. By contrast, in studies where the outcome of interest is a variable that can be measured accurately and inexpensively through a single survey question, it is unlikely that PGSs will prove valuable as control variables. In some cases, it may also be desirable to gather data on participants’ DNA after the intervention has already occurred. Such data could be used to obtain a more precise estimate of the treatment effect by controlling for the PGS. Normally, controlling for variables gathered after an intervention risks biasing the estimated treatment effect, but this concern does not apply to a variable derived from time-invariant characteristics (a participant’s DNA sequence).

Moving beyond randomized controls, confounding is always a lingering concern in observational studies. Even researchers who are fundamentally uninterested in how genes influence educational outcomes may benefit from genetic data. For example, imagine a researcher who observes that students who live in districts with better-funded schools have higher test scores than students in regions with poorly funded schools and wonders to what extent the differences are due to the causal impact of schools. Advances in genetic knowledge might eventually lead to the investigation of a set of genetic markers that can plausibly be argued to capture some of the unobserved differences in ability. Clearly, our researcher would have a much stronger case for assigning the difference in test scores to a causal impact of schools if she could show that genetic markers believed to proxy for abilities were balanced across the two school regions.

## Biological insights

To many, the primary justification for investing resources in gene-mapping efforts is that gene discoveries may implicate biological systems, thus accelerating efforts to develop effective drugs. Many medical conditions that impair learning—including dyslexia, autism spectrum disorders, epilepsy, or neurodevelopmental disorders—are substantially heritable.^38^

The GWASs of neurodevelopmental disorders conducted to date have identified only a modest number (in some cases, zero) common variants at genome-wide significance.^39–41^ But compared to height or education, these studies have been conducted in relatively small samples. The explosion in data availability in the coming years is exceedingly likely to result in a steady accumulation of associations that replicate reliably, with a qualitative pattern resembling that shown in Table 1. Studies based on exome sequencing data have shown that de novo (not inherited) rare mutations can be important causes of many neurodevelopmental disorders.^42^

The discovery of genetic associations with neurodevelopmental disorders may prove valuable for a number of reasons.

For example, they could help researchers come up with disease classifications that better reflect the underlying genetic aetiologies as opposed to shared symptoms.^43^ Imperfect understanding of such differences could conceivably sometimes cause doctors and scientists to use a single label for a diverse set of conditions that are observationally difficult to distinguish but genetically heterogeneous. In such cases, distinction between the subtypes of the disease may yield therapeutic benefits.^42^

Conversely, in some cases, there may also be cases in which substantial genetic overlap is identified between traits previously believed to have distinct genetic aetiologies.^44^ For example, the variants identified in the largest GWAS of educational attainment show evidence of enriched association with a range of neurocognitive disorders and brain function. Strikingly, genes found in previous work to harbor rare de novo (and hence rare) mutations for neurodevelopmental disorders are also statistically more likely to harbor common variants that influence normal-range variation in educational attainment.^45^

## Identification of at-risk individuals

Progress in genetic research will undoubtedly make possible, in principle, improvements in our ability to predict a range of behavioral outcomes from genetic data. For complex traits, being realistic about the amount of predictive power, we can expect with discovery samples comprising millions of individuals is important. For example, even for a highly heritable phenotype such as height, the results in Fig. 3 suggest the amount of predictive power of a PGS may soon be on par with, say, the predictive power from the average height of an individual’s biological parents. We expect genomic predictions will be possible for a range of learning outcomes and neurodevelopmental disorders.

PGSs may similarly prove valuable in research on the lifecycle development of skills, as illustrated by a recent study with extraordinarily rich phenotypic data measured in individuals tracked up to four decades.^46^ The study reported that PGSs were positively associated with reading and speaking skills in early childhood, subsequent scholastic achievement, and an index of economic security in adulthood. A mediaton analysis revealed that about half the effect of the PGS on the adult outcomes was mediated by measured cognitive and noncognitive skills. Children with higher PGSs were more likely to be raised in households with higher socioeconomic status and perhaps most intriguingly: the PGS predicted the likelihood that a child raised in a household with lower socioeconomic status would be upwardly mobile.

In some cases, knowledge of genetic risk may help parents more effectively to choose environments for their children (though mere knowledge is of limited use if no environmental intervention is available to mitigate or compensate for the risk). To borrow an example from Benjamin *et al*.^6^, if genetic screening can eventually sufficiently predict dyslexia, parents with children with substantially elevated risk for dyslexia could be given the option of enrolling their children in supplementary reading programs, years before a formal diagnosis of dyslexia.

## Conclusion

In conclusion, this perspective summarizes the current state of knowledge on the genetics of education and reviews how empirical findings should be interpreted.

Strong evidence shows genetic factors account for a substantial proportion of variation in educational attainment and its many precursors. A number of individual genes and biological pathways have been identified that underlie this variation. This biology clearly implicates the brain, often pointing to pathways shared with neurological and psychiatric disorders. Empirical results from GWASs can be used to construct genetic predictors that may have uses in experimental education studies, intervention studies, and studies of causality.

Though we believe advances in genetic knowledge in the years ahead will likely prove to have increasing utility for researchers across multiple fields, it bears repeating that the heritability of educational attainment per se is not relevant for evaluating the likely consequences of some change to education systems or policy. After all, the heritability of human height is robustly and consistently estimated to be around 80%, and yet huge secular changes in the population’s average height have occurred since industrialization.
